# Navigating the Journey in Psoriatic Arthritis: Matching the Right Patient, the Right Drug, and the Right Time

**DOI:** 10.3390/jcm14217713

**Published:** 2025-10-30

**Authors:** Ennio Lubrano, Mauro Fatica, Noemi Italiano, Fabio Massimo Perrotta

**Affiliations:** 1 Academic Rheumatology Unit, Department of Medicine and Health Sciences “Vincenzo Tiberio”, University of Molise, Via Giovanni Paolo II, C/da Tappino, 86100 Campobasso, Italy; maufat25@gmail.com (M.F.); noemiitaliano992@gmail.com (N.I.); f.perrotta85@gmail.com (F.M.P.); 2 Rheumatology, Allergology and Clinical Immunology, Department of Systems Medicine, University of Rome Tor Vergata, 00133 Rome, Italy

**Keywords:** psoriatic arthritis, disease heterogeneity, outcomes, treat-to-target, management

## Abstract

Psoriatic arthritis (PsA) is a heterogeneous, immune-mediated disease that significantly impacts quality of life, functional capacity, and healthcare systems. Over the past two decades, treatment options have expanded from conventional therapies to biologic and targeted synthetic DMARDs, enabling more effective disease control. However, many patients still fail to achieve remission or low disease activity (LDA), reflecting challenges in selecting the right treatment at the right time for the right patient. This perspective introduces a conceptual framework for PsA management using the metaphor of a journey, emphasizing three key dimensions: patient heterogeneity (“vehicle”), therapeutic options (“fuel”), and the timing of the intervention (“road”). Aligning these factors can optimize care, reduce disease burden, and improve long-term outcomes.

## 1. Introduction

Psoriatic arthritis (PsA) is a chronic, immune-mediated disease that commonly occurs in individuals with psoriasis (PsO) [1]. Its clinical presentation is remarkably heterogeneous, encompassing peripheral arthritis, axial disease, enthesitis, dactylitis, skin and nail involvement, as well as extra-musculoskeletal manifestations [2]. Moreover, PsA is associated with multiple comorbidities such as cardiovascular disease, metabolic syndrome, and fibromyalgia, all of which can negatively impact its course and further complicate its management [3].

The burden of PsA may extend beyond what can be objectively assessed in clinical practice. Patients often face reduced quality of life, functional impairment, chronic pain, and fatigue [4]. As a result, PsA can profoundly affect work productivity and social participation while driving up high direct and indirect healthcare costs, including hospitalizations, specialist visits, and long-term disability [5]. These challenges underscore the need for treatment strategies that not only control inflammation but also address the broader consequences of the disease.

Over the past two decades, PsA treatment has undergone a profound transformation. Historically, therapeutic options were limited to non-steroidal anti-inflammatory drugs (NSAIDs), corticosteroids, and conventional synthetic disease-modifying antirheumatic drugs (csDMARDs) such as methotrexate [6]. While these agents provide symptomatic relief, they often fail to prevent joint damage or achieve long-term disease control. The advent of biologic DMARDs (bDMARDs)—beginning with tumor necrosis factor (TNF) inhibitors and later expanding to interleukin (IL)-17 inhibitors, IL-12/23 inhibitors, and IL-23 inhibitors—marked a revolutionary shift [7]. More recently, targeted synthetic DMARDs (tsDMARDs), including Janus kinase (JAK) inhibitors and phosphodiesterase-4 (PDE4) inhibitors, have further expanded the therapeutic landscape [8]. With these advancements, clinicians now have an unprecedented array of powerful tools to manage PsA and strive for the achievement of the lowest possible level of disease activity in all domains of disease. In this context, the treat-to-target (T2T) strategy has improved PsA management: by setting remission or low disease activity (LDA) as a clear therapeutic goal, clinicians are encouraged to monitor patients systematically and adjust therapy accordingly [9].

In this context, due to the multifaceted nature of the disease, different clinical indices that capture disease activity and disease state were validated to be used in clinical trials and real-world settings, with the aim of standardizing PsA assessment [10,11,12]. Table 1 summarized the features of the main scores.

Randomized controlled trials and international recommendations have validated this approach, demonstrating improved outcomes compared with routine care [13,14].

Despite the recent therapeutic breakthroughs and strategies, real-world data reveal a sobering reality: a substantial proportion of patients do not achieve or sustain remission or LDA, and many continue to experience flares, progressive structural damage, and diminished quality of life [15]. This disconnection between the potential of modern therapies and actual patient outcomes highlights persistent gaps in care. Why does this gap persist? Probably, the problem is not a lack of options, but rather how we navigate them. Clinicians must decide which drug to prescribe, when to start or escalate therapy, and how to account for a patient’s comorbidities, disease phenotype, and preferences.

As a result, the current landscape of PsA management resembles a crowded highway at rush hour, where countless vehicles move in the same direction but on different paths, at different speeds, and with varying degrees of efficiency. Each patient embarks on this journey in a unique vehicle, with different engines, fuel capacities, and road conditions. Even with the same destination, the experience of travel can be radically different: some patients advance steadily on a clear route, while others struggle through stop-and-go traffic, forced to navigate obstacles, diversions, and periods of frustrating standstill. Just as a skilled driver needs the right vehicle, the right fuel, and the right route to reach their destination timely and safely, PsA care must align three crucial dimensions: the right patient, the right drug, and the right time. Accordingly, we will use this metaphor as a conceptual roadmap to guide the discussion of PsA management, illustrating how aligning these three aspects can optimize the journey toward remission or LDA.

This article is based on a narrative literature review. Relevant studies were identified through PubMed and Scopus using combinations of the terms psoriatic arthritis, treatment, biologic DMARDs, targeted synthetic DMARDs, precision medicine, and treat-to-target. Additional references were retrieved from the bibliographies of key papers. Only peer-reviewed English-language publications from January 2000 to June 2025, including pivotal clinical trials, were considered, while conference abstracts and non–peer-reviewed materials were excluded.

## 2. The Patient as the Vehicle

Each individual with PsA travels in a different type of vehicle. Understanding the patient’s “vehicle type” is essential for anticipating therapeutic needs and potential barriers to success. In 1973 the classification provided by Moll and Wright described five clinical subtypes: asymmetric oligoarthritis, symmetric polyarthritis, distal interphalangeal joint–predominant arthritis, spondylitis, and arthritis mutilans [16]. While historically important and still relevant, this classification emphasizes anatomical features but offers a partial view of the evolving and often overlapping patterns of PsA in clinical settings.

Over the past decade, there has been a paradigm shift toward domain-based classification, recognizing that PsA may involve a combination of peripheral arthritis, axial disease, enthesitis, dactylitis, and skin or nail disease in varying degrees and at different timepoints [13]. Data from cluster and machine learning analyses suggest that these domains often group into two to four major phenotypes, predominantly peripheral arthritis, predominantly axial disease, skin/enthesitis-dominant and mixed phenotypes [17]. Importantly, these phenotypes are not static: patients may shift “lanes” over time, with new domains emerging or previous one regressing, underscoring the need for flexible, adaptive management strategies. Beyond clinical domains, a growing body of research points to the existence of biological endotypes, subgroups defined by distinct molecular and immunological signatures [18]. Transcriptomic and proteomic studies have highlighted heterogeneity within PsA synovium and skin, with varying degrees of IL-23/IL-17 axis activity, TNF-driven pathways, and other inflammatory pathways [19]. Moreover, peripheral T-lymphocytic phenotyping might be a useful tool for precision medicine approach, by allocating treatment choices based on the activation and polarization of T lymphocytes in peripheral blood, with the aim to targeting a specific immune-phenotype [20]. These biological signatures may eventually provide predictive insights into which patients will respond to specific therapies. In this context, emerging tools such as synovial tissue biopsy hold promise for refining patient stratification and guiding precision therapy [21]. However, in current practice, these tools are still largely investigational, and treatment decisions rely primarily on clinical observation rather than mechanistic profiling.

To sum up, each patient can be seen as a “vehicle” traveling toward remission or LDA. Some resemble high-performance sports cars—young, with few comorbidities and high treatment tolerance—who may benefit from rapid escalation to highly effective biologic or targeted therapies to achieve early disease control. Others are more like heavily loaded trucks, burdened by conditions such as obesity or cardiovascular disease, requiring a slower, more cautious approach that also prioritizes safety and comorbidity management [22]. Many patients fall between these extremes, with characteristics that evolve over time like hybrid vehicles adapting to changing road conditions, underscoring the need for flexible and dynamic treatment strategies. This metaphor reinforces that no single management pathway fits all patients and highlights the importance of tailoring therapeutic decisions to individual profiles to optimize outcomes. In summary, the “right patient” dimension highlights the key role of understanding who is being treated. Future research should focus on linking phenotypes and endotypes to therapeutic outcomes, moving toward a precision medicine model where the vehicle is clearly identified before selecting the fuel and mapping the route. Only by refining our ability to classify and predict can we ensure that each patient’s journey toward remission is as efficient and effective as possible.

## 3. The Drug as the Fuel

Even the best vehicle cannot move without the right fuel. The expanding landscape of bDMARDs and tsDMARDs provides a range of “fuel types,” each with unique mechanisms of action and indications. TNF inhibitors were the first class of targeted therapies to transform PsA management, acting by this pivotal cytokine driving synovial inflammation, entheseal pathology, and structural joint damage [23]. Subsequent therapies expanded the range of immunological targets. IL-12/23 inhibitors, such as ustekinumab, block the shared p40 subunit, thereby modulating both Th1 and Th17 immune pathways involved in PsA pathogenesis [24]. More recently, selective IL-23 inhibitors, including guselkumab and risankizumab, have been developed to specifically target the p19 subunit of IL-23, providing more focused downstream suppression of Th17-driven inflammation, which plays a key role in joint and skin disease [25]. The IL-17 pathway has become a key therapeutic target, with agents like secukinumab and ixekizumab neutralizing IL-17A, bimekizumab blocking both IL-17A and IL-17F for broader modulation, and the receptor antagonist brodalumab inhibiting signals from IL-17A, IL-17C, and IL-17F [26]. Beyond biologics, tsDMARDs have expanded treatment options: JAK inhibitors (e.g., tofacitinib, upadacitinib) act intracellularly by interfering with the JAK-STAT signaling cascade, a pathway downstream of multiple pro-inflammatory cytokines [27], whereas the PDE4 inhibitor apremilast regulates intracellular cyclic AMP levels, exerting indirect anti-inflammatory effects [28]. Recently, early results from a phase III study showed the efficacy and safety of deucravacitinib, a TYK2 inhibitor, which modulates the intracellular signaling mainly coming from IL-23 receptor, providing an alternative method to block Th17 polarization [29]. Other promising drugs include nanobodies, such as sonelokimab, and affibodies, such as izokibep, which offer highly specific targeting and improved tissue penetration, making them attractive options for future therapeutic strategies for PsA [30,31].

Modern PsA management follows a domain-based approach, as endorsed by both Group for Research and Assessment of Psoriasis and Psoriatic Arthritis (GRAPPA) and European Alliance of Associations for Rheumatology (EULAR) recommendations, which emphasize aligning therapeutic choices with the patient’s predominant clinical features while also considering comorbidities and patient preferences [13,14]. This strategy reflects the heterogeneous nature of PsA and supports selecting agents whose mechanisms best target the manifestations causing the greatest burden, recognizing that disease activity and priorities may shift over time and that multidisciplinary approach is often required, especially when extra-musculoskeletal manifestations or comorbidities are present. The role of combination therapy with csDMARDs remains nuanced. While methotrexate is often used in early disease or as background therapy, randomized trials have shown limited additional benefit when combined with bDMARDs and tsDMARDs [32].

Safety is an equally critical dimension in therapeutic decision-making, with profiles differing across drug classes. While overall tolerability is generally favorable, all these agents carry a risk of infections, most commonly mild upper respiratory tract infections. In this regard, TNF inhibitors are associated with latent tuberculosis reactivation, IL-17 inhibitors with mucocutaneous Candida infections, and JAK inhibitors with herpes zoster [33]. Moreover, JAK inhibitors require monitoring for thromboembolic and cardiovascular events, while PDE4 inhibition with apremilast may be associated with mild gastrointestinal symptoms and weight loss [34].

To sum up, PsA care requires balancing efficacy by domain with safety and comorbidity considerations, while remaining flexible as the disease evolves. Although several classes of bDMARDs and tsDMARDS are now available, there are no robust biomarkers or predictive algorithms to guide the initial choice [35]. As a result, therapeutic decisions often follow a trial-and-error pattern, with patients cycling through multiple agents over time. Lastly, it is crucial that the “fuel” must be replenished consistently to ensure the vehicle continues toward its destination. Adherence and persistence with prescribed therapy are critical, as lapses or discontinuations can lead to disease flares, progression of joint damage, and loss of previously achieved improvements. Therefore, optimizing treatment involves patient education, close follow-up, and proactive approach to potential logistical barriers.

## 4. Time as the Road Conditions and Signals

Even a well-matched vehicle and fuel will struggle on a bumpy road. In PsA care, time plays a dual role: it shapes the road conditions, determining whether the journey begins on a smooth highway or a rough, obstacle-filled path, and it provides the traffic lights and signals that guide when to accelerate, change course, or hold steady. Both elements are critical, since poor initial conditions slow progress, while misreading signals can lead to wrong turns or delays. In this context a fundamental aspect of PsA care is early diagnosis. Population-based studies indicate that many patients experience months or even years between symptom onset and a confirmed diagnosis [36]. This diagnostic delay exposes patients to prolonged inflammation, leading to increased risk of structural damage, impaired function, greater comorbidity burden, and a more hazardous journey which may limit the potential benefit of advanced therapies [4]. Patients diagnosed early are more likely to achieve substantial gains from treatment, emphasizing the critical impact of timely recognition [37]. In fact, intervention studies suggest there is a “window of opportunity” during which prompt advanced treatment can significantly alter the natural course of PsA [38].

However, this window can be easily missed in routine practice and there is no current standardized definition of what constitutes “early” PsA. For instance, in the TICOPA study, early PsA was defined as symptom duration of less than 24 months [39], whereas the FOREMOST trial used a broader cut-off of ≤ 5 years [40], reflecting variability across studies. To improve early detection, a recent Delphi consensus proposed standardized terminology for PsA development phases: (I) individuals at increased risk based on clinical or genetic factors but without symptoms; (II) individuals with subclinical imaging abnormalities indicating silent inflammation; (III) individuals with musculoskeletal symptoms not explained by other diagnoses [41]. These stages—at risk, subclinical, and early PsA—are central to research and interception strategies. Recognizing early warning signs is especially important in patients with PsO, who form the main at-risk population. Medium- to long-term risk factors for PsO-to-PsA transition include nail involvement, obesity, severe PsO, and family history of PsA, reflecting genetic and systemic susceptibility [42]. Short-term predictors, which may indicate imminent disease onset, include arthralgia and subclinical imaging-detected inflammation, such as sonographic entheseal changes [42].

Recognizing these factors can help clinicians to identify patients at risk and facilitate closer monitoring or early intervention to potentially delay or prevent PsA onset [43]. The concept of PsA prevention is highly debated.

From a pathogenetic perspective, the inhibition of key cytokines such as IL-23, which drive the activation of resident memory T cells, potentially disrupting the immunological cascade responsible for synovial and entheseal inflammation. Observational data suggest that early systemic or biologic therapy for psoriasis may reduce the risk of subsequent PsA compared with phototherapy, topical agents, or csDMARDs [44,45,46,47,48,49].

However, these findings must be interpreted cautiously, as prospective trials specifically assessing PsA prevention are lacking [50]. Current evidence therefore remains inconclusive, and it is uncertain whether these agents truly prevent PsA or merely delay its onset, leaving open the question of their disease-modifying potential in the preclinical phase. Beyond early diagnosis and interception, timely treatment adaptation remains crucial. Over the disease course, patients may face road hazards like potholes, detours, and sharp curves, representing disease flares, treatment-related adverse events, and the new onset of comorbidities. In this context, clinical, laboratory, and imaging evaluations over time are the signals that guide when to accelerate, maintain course, or change therapy. Within a T2T framework, these signals include regular assessment of composite disease activity indices, such as achieving remission or LDA within 3–6 months of therapy initiation or escalation [9]. Failure to meet these milestones indicates primary inefficacy, while subsequent disease worsening after initial response indicates secondary loss of efficacy, both prompting therapeutic adjustments [13]. Additionally, new comorbidities or treatment-related adverse events serve as critical stop signs, requiring either dose modification or a switch in drug class [51]. Systematic timely monitoring, such as the 12-week evaluations used in TICOPA trial, ensures these signals are detected promptly, preventing unnecessary delays or premature treatment changes [39]. Therefore, a crucial challenge is striking this balance: recognizing primary inefficacy or loss of response early enough to adjust treatment, control inflammation and prevent structural damage, while avoiding excessive switching that disrupts stability, increases costs, and risks overtreatment. Despite this, uncertainty often remains in clinical practice about when and how to escalate therapy, whether by optimizing symptomatic treatment, adjusting dosages, or switching mechanisms of action [52].

In this context, recent real-world and registry data have compared cycling within the same biologic class and swapping to a different mechanism of action in PsA. While both strategies generally showed comparable effectiveness and drug survival, a trend toward better outcomes with swapping was observed in cases of primary non-response, emphasizing that sequencing decisions should be individualized based on the reason for treatment failure and patient characteristics [53,54,55]. Moreover, factors as the presence of comorbidity (obesity, metabolic syndrome, fatty liver disease or history of infections or neoplasms) may have an important role in shaping treatment decision in both naïve or biologic experienced patients [3].

Ultimately, effective PsA management requires clinicians to navigate both the conditions and the signals of the therapeutic journey. Early recognition and intervention can smooth the road ahead, while careful interpretation of disease dynamics ensures that each move is made at the right time. Balancing these aspects is crucial for achieving stable, long-term disease control and keeping the patient’s journey on track.

## 5. Conclusions

**Figure 1:**
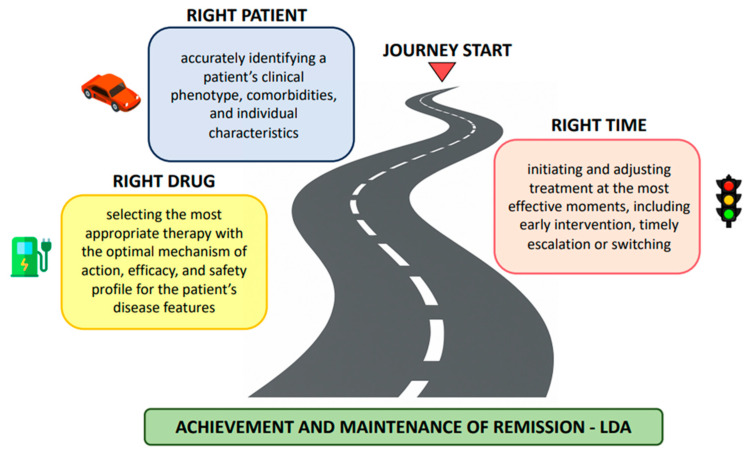
Navigating Psoriatic Arthritis (PsA) management. Schematic representation of PsA. management as a journey toward remission or low disease activity (LDA), guided by three interconnected dimensions: the right patient (understanding disease heterogeneity), the right drug (selecting optimal therapy), and the right time (early and timely intervention).

The journey to remission or LDA in PsA is no longer blocked by a lack of therapeutic options. The current challenge lies in navigation: selecting the right vehicle (understanding patient heterogeneity), providing the right fuel (choosing the most appropriate and sustainable therapy), and traveling on the right road (acting at the optimal time and adjusting course when needed). This conceptual roadmap is illustrated in Figure 1. Paradigmatic cases of patient journey are illustrated in Box 1.

Box 1 **Paradigmatic cases of patient journey in real clinical practice.**

***Case 1***

A 39-year-old man with a 15-year history of psoriasis, diagnosed at age 24, developed arthritis of the left knee and dactylitis of the third and fourth fingers of the right hand. No comorbidities or extra-articular manifestations were present.

A diagnosis of PsA was established in 2020, and the patient started treatment with methotrexate 15 mg once weekly and non-steroidal anti-inflammatory drugs.

After six months, disease activity remained high, with a DAPSA score of 25.2 and a BSA of 4%, including scalp and nail psoriasis.

Following infectious disease screening, the patient initiated ixekizumab 80 mg every four weeks.

At the 3-month follow-up, a marked reduction in disease activity was observed, with complete clearance of skin lesions and achievement of DAPSA remission.

After five years of follow-up, the patient maintained sustained remission, allowing extension of the ixekizumab dosing interval.

***Case 2***

A 67-year-old woman with severe psoriasis diagnosed at age 51 presented with polyarthritis involving the knees, elbows, and interphalangeal joints of the feet. Her past medical history included type 2 diabetes mellitus, hypertension, obesity (BMI > 30), and hyperuricemia.

She was initially treated with methotrexate 15 mg weekly, followed by adalimumab 40 mg every two weeks, but showed no clinical improvement after six months. Methotrexate was discontinued due to gastrointestinal intolerance.

After six months of adalimumab therapy, her DAPSA score was 34.2 and BSA 7%.

She was then switched to secukinumab 300 mg monthly, achieving marked improvement in cutaneous psoriasis (BSA reduced from 7% to 0%) but persistent musculoskeletal disease activity (DAPSA = 28.5, not reaching minimal disease activity) after one year.

Subsequently, treatment with ixekizumab was initiated, but discontinued after three months because of recurrent injection-site reactions.

At her last visit, the patient started guselkumab 100 mg every 8 weeks, achieving clinical improvement. However, at radiographic assessment, presence of both new bone formation and articular erosions at interphalangeal joints and osteophitis at knee were present, further complicating the clinical course.

In this regard, a few years ago, we proposed the term “Psoriatic Syndrome” to better capture this concept, emphasizing a holistic view that integrates the diverse clinical features of PsA into a unified framework [56]. This new concept embraces PsA pathogenesis as a potential sequence of events which can or cannot happen (“run together”) in the same patient and aimed to propose a pathway to follow for a more strategic disease management, considering the target to be treated and the patient characteristics. With coordinated efforts across healthcare systems, we moved the contents of Box 1 into the text box. Please confirm that clinical practice, and research priorities—which are summarized in Box 2—are needed to achieve this vision.

Box 2 **Key Research Priorities in Psoriatic Arthritis (PsA).**

- Enhance early detection and intervention—Define preclinical stages and strategies to reduce diagnostic delays.
- Link phenotypes and endotypes to therapy response—Identify molecular and clinical signatures predictive of treatment outcomes.
- Develop decision-support tools—Integrate clinical, imaging, biomarker, and patient-reported data to guide individualized therapy.
- Leverage real-world evidence and registries—Monitor long-term outcomes and optimize T2T strategies.

In conclusion, the achievement of remission or LDA is a continuous journey, requiring flexibility as disease manifestations shift over time. By systematically aligning who we treat, when we act, and how we select and adapt therapies, we can transform PsA care from trial-and-error driving into a coordinated, high-speed route toward these lasting outcomes.

## Figures and Tables

**Table 1:** Main Psoriatic Arthritis Disease Activity Indices used in clinical trials and clinical practice.

Index	Components/Formula	Cut-Offs for Disease Activity States	Pros/Cons	Monitoring
**DAPSA** (Disease Activity index for PSoriatic Arthritis)	DAPSA = TJC(68) + SJC(66) + PtGA (0–10 VAS) + Pt Pain (0–10 VAS) + CRP (mg/dL or mg/L)	**Remission:** ≤4 **Low:** >4–14 **Moderate:** >14–28 **High:** >28	**Pros: Quick,** feasible, easy to use, sensitive to change **Cons:** do not include skin/nail, enthesitis, axial disease and dactylitis. Cut-offs based on physician evaluation	**3–6 months**
**cDAPSA** (Clinical DAPSA)	Same as DAPSA but **excludes CRP:** cDAPSA = TJC(68) + SJC(66) + PtGA + Pt Pain	**Remission:** ≤4 **Low:** >4–13 **Moderate:** >13–27 **High:** >27	**Pros:** Quick, feasible, easy to use, sensitive to change **Cons:** do not include skin/nail, enthesitis, axial disease and dactylitis. Cut-offs based on physician evaluation. Absence of laboratory parameters	**3–6 months**
**PASDAS** (Psoriatic Arthritis Disease Activity Score)	Composite index combining multiple domains: **Formula:** PASDAS = (0.18 × √Physician Global VAS) + (0.159 × √PtGA VAS) + (0.253 × √SF-36 PCS) − (0.101 × √SJC66) − (0.048 × √TJC68) + (0.23 × ln(Swollen Entheses + 1)) + (0.37 × ln(CRP + 1)) + 0.102 Range ≈ 0–10.	**Remission:** ≤1.9 **Low:** >1.9–3.2 **Moderate:** >3.2–5.4 **High:** >5.4	**Pros:** accounts for nearly all disease for most people. Developed on real life treatment decision. Disease state based on Physician and patient opinion. **Cons:** Bit slower. Need SF-36. Need online calculator	**3–6 months**
**MDA** (Minimal Disease Activity)	Binary outcome (Yes/No). MDA = achieved if **≥5 of 7** criteria met: TJC ≤ 1; SJC ≤ 1; PASI ≤ 1 or BSA ≤ 3%; Patient Pain on VAS ≤ 15 mm; PtGA VAS ≤ 20 mm; HAQ-DI ≤ 0.5; Tender entheseal points ≤ 1	**MDA achieved:** ≥5/7 criteria met **Non-MDA:** <5/7	**Pros:** Quick and feasible. Easy to calculate. Correlates with patient opinion. Account for nearly all disease domains **Cons:** Only a measure of disease state. Could allow active joint disease. Absence of laboratory parameter	**3–6 months**

## Data Availability

No new data were created or analyzed in this study.

## Abbreviations

The following abbreviations are used in this manuscript:

bDMARD	Biologic disease-modifying antirheumatic drug
csDMARD	Conventional synthetic disease-modifying antirheumatic drug
DAPSA	Disease Activity in PSoriatic Arthritis score
EULAR	European Alliance of Associations for Rheumatology
GRAPPA	Group for Research and Assessment of Psoriasis and Psoriatic Arthritis
IL	Interleukin
JAK	Janus kinase
LDA	Low disease activity
MDA	Minimal disease activity
NSAID	Non-steroidal anti-inflammatory drug
PDE4	Phosphodiesterase-4
PsA	Psoriatic arthritis
PsO	Psoriasis
T2T	Treat-to-target
TNF	Tumor necrosis factor
